# CBCT-Based Morphological Study of the Accessory Foramina of the Canalis Sinuosus: Prevalence, Morphological Variants, and Significance for Implant Surgery

**DOI:** 10.3390/jcm14041083

**Published:** 2025-02-08

**Authors:** Sigmar Schnutenhaus, Christian Heckemann, Werner Götz, Constanze Olms

**Affiliations:** 1 Center of Dentistry, Department of Prosthetic Dentistry, Ulm University Hospital, 89081 Ulm, Germany; 2 Center for Dental Medicine Prof. Dr. Schnutenhaus, 78247 Hilzingen, Germany; 3 Laboratory for Oral Biology, Center for Oral and Maxillofacial Medicine, University of Bonn, 53111 Bonn, Germany; 4 Private Dental Practice of the Specialist Dentists Olms, 29410 Salzwedel, Germany

**Keywords:** cone-beam computed tomography, maxillary sinus, canalis sinuosus, anterior maxillary alveolus, accessory canal, anterior superior nerve

## Abstract

**Objective:** The canalis sinuosus in the premaxillary region often has accessory canals palatal to the central and lateral incisors. These small anatomical structures are of increasing interest due to numerous case reports of postoperative complications following surgery in the upper anterior region. **Methods:** This study examined the number, position, and extent of the accessory foramina of the canalis sinuosus in 210 patients. Furthermore, this study examined the distances to neighboring teeth and to the buccal cortical bone in edentulous patients. Three-dimensional tomographic (CBCT) images were created with a resolution of 0.2 voxels and were evaluated using the Osirix MD 11.0 program. **Results:** The results showed a prevalence of 97% for accessory foramina, confirming them as a clear anatomical structure. Males had significantly more terminal openings of the canalis sinuosus than females, with clustering in the fourth to sixth decades of life. The foramina had a mean extension of 0.9 mm and a mean distance of 4.6 mm to adjacent teeth. **Conclusions:** This narrow position is particularly relevant for surgical procedures. Greater attention should be focused on larger foramina in implant planning, as postoperative complications are increasingly being described. Foramina and accessory canals should be detected in three-dimensional, navigated implant planning in order to minimize the risk of injury during oral surgery.

## 1. Introduction

The canalis sinuosus, first described by Jones in 1939, is a frequently tortuous anatomical structure that has been demonstrated in 88% to 100% of patients [1,2]. This structure is increasingly recognized as a relevant anatomical unit and is no longer considered merely a variant.

As a rule, the sinuosal canal emerges approximately 25 mm behind the infraorbital foramen on both sides, runs along the lateral nasal wall to the orbital floor, and continues to the anterior nasal opening, often ending with small accessory canals to the palatal vault [3,4]. These foramina are often present palatal to the maxillary incisors and canines and are found in 15.7% to 100% of patients [2,3,5,6,7,8,9]. The average diameter of the accessory canals is 0.5–2.0 mm [2,5,8]. A terminal branch of the superior anterior alveolar nerve from the infraorbital nerve runs together with the artery of the same name in this canal.

The arterial structure of the canalis sinuosus is a small branch of the infraorbital artery, which, like the nasopalatine artery, originates from the important maxillary artery in the maxilla [3,4]. These arteries supply the maxillary anterior teeth and surrounding soft tissue, with anastomoses also playing a role [10].

There is a significant anatomical difference between the incisive canal and the nasopalatal duct [10]. The latter develops during the prenatal development phase, more precisely during the 8th embryonic week, from epithelial remnants within the incisive canal. From the 13th to 14th week of embryonic development, the latter forms a continuous connection between the oral and nasal cavities in the form of an epithelial cord [11].

Shortly before birth, the nasopalatine duct finally regresses, leaving only an obliterated epithelial remnant in adults. Further into the course of development, the incisive canal unites in a caudal direction in a Y-shape and opens at the center of the palatine behind the incisive papilla into the incisive foramen. Nerves, as well as various blood vessels, run within the incisive canal. The main arterial structure here is the nasopalatine artery, which is considered to be the terminal branch of the sphenopalatine artery. This artery forms an anastomosis with the major palatine artery as it passes through the incisive canal to its supply area, the hard palate (palatum durum). The veins running parallel to it have equivalent names.

The mucosa of the hard palate is innervated by the nasopalatinal nerve, which originates from the maxillary nerve as the largest terminal branch of the posterior superior nasal branches and passes through the incisive foramen. In addition to the sensory supply of the palatal region of the maxillary anterior teeth, the major palatine nerve, another branch of the maxillary nerve, has additional branches that contribute to sensory innervation. As with any surgical procedure, injury to neighboring structures such as blood vessels, nerves, and tissues can lead to relevant complications and postoperative restrictions. Postoperative restrictions after implantation in the upper jaw can be caused particularly by injuries to nerves or vessels that were unintentionally damaged during the procedure. Damage to nervous structures can result in numbness, tingling, or even persistent sensory disturbances in these areas. In most cases, these complaints are temporary and subside within a few weeks or months, but in rare cases, permanent restrictions may remain.

Another possible complication is vascular injury. Such vascular injuries can result in increased postoperative bleeding or hematomas, which not only delay wound healing but can also increase pain and swelling. Hemorrhagic events, as well as hypanesthesia and anesthesia in the implantation area, occur frequently, especially in implantations in the premaxilla [12]. However, thanks to progressive developments in three-dimensional imaging, computer-assisted planning, and template-supported implantations, these complications have become significantly less frequent.

Nevertheless, implantations in the maxilla remain more risky than in the mandible. Gaeta-Araujo et al. found that the prevalence of perforations of adjacent structures, such as the maxillary sinus or the incisive canal, is 43.5% in the maxilla, while only 11.3% of cases are affected in the mandible [13]. The limited space available in the maxilla is a significant factor here [14]. Despite technical advances, complications continue to occur, often affecting the sensory system in the surgical area. Damage to the nasopalatine nerve was long thought to be the cause of hypoesthesia and anesthesia, but more recent studies have refuted this assumption [6,7].

Implant placement should always be planned with the final prosthetic restoration in mind, taking into account both functional and aesthetic aspects [15]. For this reason, the use of imaging techniques such as digital volume tomography (CBCT) is essential today in order to achieve predictable outcomes [16]. Orhan et al., Ghandourah et al., and Tomrukcu et al. showed that two-dimensional orthopantomography (OPG) is not sufficient for assessing small anatomical structures such as the accessory canals of the sinuosal canal [5,7,9]. These can easily be mistaken for periapical lesions, which are exacerbated by radiologic artifacts such as superimposition and image distortion. Therefore, a three-dimensional image should also be obtained during postoperative diagnostics [5,17]. With modern CBCT technologies and powerful software applications, the precise visualization of anatomical conditions and exact surgical planning are now possible.

The aim of this study was to examine the extent to which the canalis sinuosus is present in a southern German population and whether there are significant correlations with the age or gender of patients. In addition, whether there are differences in the number, localization, diameter, and distances to neighboring anatomical structures was investigated. The results should allow conclusions to be drawn for diagnostics and preoperative planning prior to implant surgery.

## 2. Materials and Methods

The present monocentric, retrospective study with randomly selected patients was approved by the Ethics Committee of the State Medical Association of Baden-Württemberg (AZ F-2020-024-z, approval on 3 March 2020) prior to the start of the study. The study design was developed based on the STROBE checklist [18].

CBCT images of 210 patients from a dental clinic in Hilzingen (Germany) between February 2009 and July 2019 were used to analyze the accessory canals of the canalis sinuosus. The DICOM datasets were anonymized and evaluated retrospectively.

The patients were divided into three age groups: under 41 years (group 1), 41 to 60 years (group 2), and over 61 years (group 3); there were 35 women and 35 men per group. The classification of patients into three age groups was carried out to analyze age-dependent differences in the examined parameters. This classification allows for a structured evaluation of potential age-related effects and contributes to the comparability of the results. Commonly used categories in clinical studies (<41 years, 41–60 years, >61 years) were used to distinguish possible influencing factors related to age. Inclusion criteria were complete CBCT images of the maxilla and nasal floor; exclusion criteria included pathological changes such as apical radiolucencies at root tips, cysts or radiologically hypodense zones of unknown origin, implants in the area of the canalis sinuosus, and metallic dental restorations. If further limitations—such as atypical anatomical variations, superimposition with other structures, or insufficient image quality of the CBCT—were detected, the affected patients were excluded from the analysis. CBCT images were taken using the Gendex GXCB-500 CBCT scanner (Gendex Dental Systems, Hatfield, PA, USA), with patients positioned according to established standards. The scan time was 23 s with a resolution of 0.2 mm.

CBCT data were loaded into i-CAT VisionTM software (version 1.9.3.13) and then transferred to Osirix MD (version 11.0) for analysis. The measurements were performed on a calibrated monitor in a darkened room.

The analysis began with a descriptive mapping of the diameters of the accessory foramina in the axial and sagittal planes, followed by the measurement of the distances of the foramina to neighboring teeth. These measurements helped to identify possible conflicts with future implant positions. The reference value for implant positions was 10 mm from the alveolar ridge.

**Figure 1:**
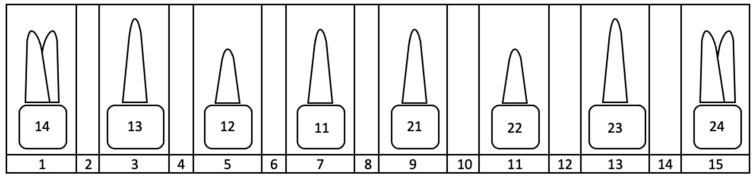
Overview of the coding of the foramen position using numbers 1–15 listed in the bottom line of the diagram. Position 1 corresponds to the mediopalatal position at tooth 14, and position 15 corresponds to the mediopalatal position at tooth 24. The even numbers represent the approximal position between two teeth in each case.

For the measurements, all foramina between the first premolars (teeth 14–24) were taken into account in the axial plane. The foramen to be measured was coded in the axial plane using an assigned number (Figure 1).

Only clearly recognizable foramina were measured, whereby the incisive foramina were excluded. The largest diameters of the foramina were recorded in the axial and sagittal planes. In the sagittal plane, the largest caudal diameter was measured if the canal wall was clearly visible.

**Figure 2:**
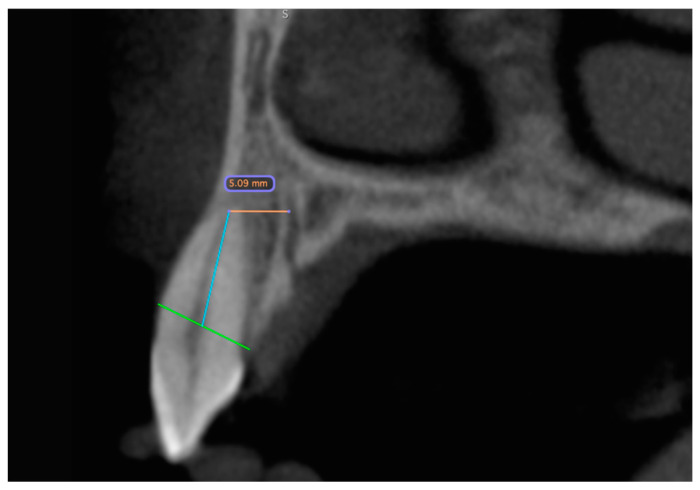
CBCT dataset with the distance measurement of the accessory canal to the neighboring tooth shown in the sagittal slice. The green line represents the bone level. Starting from this, a blue line is drawn along the wall of the pulp cavity to the apex of the tooth. At its end, it forms an endpoint of the orange measurement section. The distance is measured from here to the outer edge of the canal.

**Figure 3:**
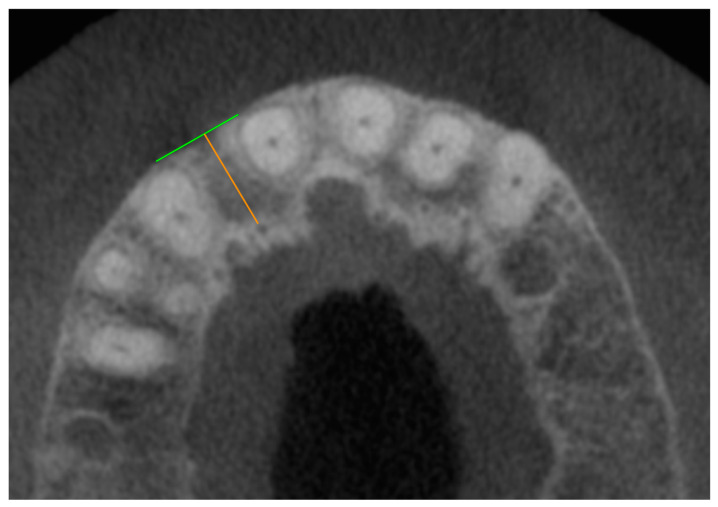
CBCT dataset with the axial representation of an accessory foramen of the canalis sinuosus palatal to missing tooth 12. The green line forms a clearly recognizable outer cortex of the alveolar ridge as an endpoint of the orange measuring section, which begins at the outer edge of the foramen.

In the second step, the distances between the foramina and the neighboring teeth were measured (Figure 2). If no tooth was nearby, the alveolar cortex was used as a reference (Figure 3). The measurement of the distance of the foramina to the neighboring tooth and the buccal outer cortex of the alveolar ridge was deliberately chosen because it allows a direct reference to the clinically preferred region for implant placement. The aim of this measurement was to establish the anatomical reference to the relevant structures in order to ensure the precise and safe placement of the implant. On the one hand, the implant is usually inserted with respect to the tooth and prosthetically oriented palatal to the original tooth position in order to ensure optimal prosthetic restoration. On the other hand, the palatal positioning and angulation of the implant are carried out deliberately to maintain a sufficient bone layer circumferentially around the implant, which is essential for long-term stability and osseointegration. If the tooth was not clearly visible, the value was set to 0.

As a supplementary measure to the listed examinations, exploratory evaluations were also conducted using datasets from an additional 109 patients, each containing records from two different time points. The time intervals between these individual datasets varied considerably in some cases. To ensure better comparability, only images from male subjects with a time interval similar to that of the corresponding female patients were selected for inclusion in this study. The intervals between the different imaging dates ranged from more than five years to over nine years. Based on the time elapsed between the radiographic images, patients were categorized into five distinct groups. Following the application of these selection criteria, a total of ten cases were chosen from the subject pool, with each of the five groups including datasets from one female and one male patient for examination. Group 1 comprised patients in whom the time interval between the images was 61.9 months (female) or 62.9 months (male). The second group was assigned imaging intervals of more than six but less than seven years. In the third group, imaging intervals were 85.6 months (female) and 86.4 months (male). In cohort four, the time difference between the datasets was the same for both sexes at 97.5 months. The fifth group of X-rays was taken at an interval of more than nine years.

For this subgroup, two CBCT images were available from different time points. The methodological approach mirrored that of the first part of the evaluation. The objective of this section of the study was to analyze potential changes in the size of the foramina over time within individual patients.

The data collected for each of the 210 plus 10 patients were recorded for statistical analysis in the spreadsheet program Excel 2019 for Mac version 16.70 (Microsoft Corporation, Redmond, WA, USA). For the statistical evaluation, the data were analyzed with SPSS version 26 (IBM Corp., New York, NY, USA). The mean values, standard deviations, and confidence intervals were calculated. Both parametric (ANOVA) and non-parametric (Mann–Whitney U test) tests were used. A significance level of 5% was applied. All results were interpreted to confirm the hypothesis.

## 3. Results

The main objective of this study was to analyze the accessory canals of the canalis sinuosus with regard to their position, diameter, and distance to adjacent teeth, especially with regard to possible subsequent implant placement after tooth loss in the anterior maxilla.

### 3.1. Data Analysis

To investigate the accessory canals of the canalis sinuosus, the data of 210 patients were evaluated in a descriptive and explorative analysis. These were divided into three age-dependent groups of 70 patients each:

Group 1: participants under 41 years of age (average age of 32.4 years);

Group 2: participants aged 41 to 60 years (average age of 50.5 years);

Group 3: participants over 60 years old (average age of 68.7 years).

The youngest study participant was 14 years old, and the oldest was 79 years old. Overall, the gender distribution was balanced, with 105 men and 105 women (50%).

### 3.2. Analysis of Duct Localization, Duct Quantities, and Diameter

In the 210 cases, the number, position, and diameter of the accessory foramina of the canalis sinuosus were analyzed. A total of 555 foramina were identified:

- Group 1: 180 foramina (66 patients, 32.4%);
- Group 2: 200 foramina (69 patients, 36.1%);
- Group 3: 175 foramina (69 patients, 31.5%).

**Figure 4:**
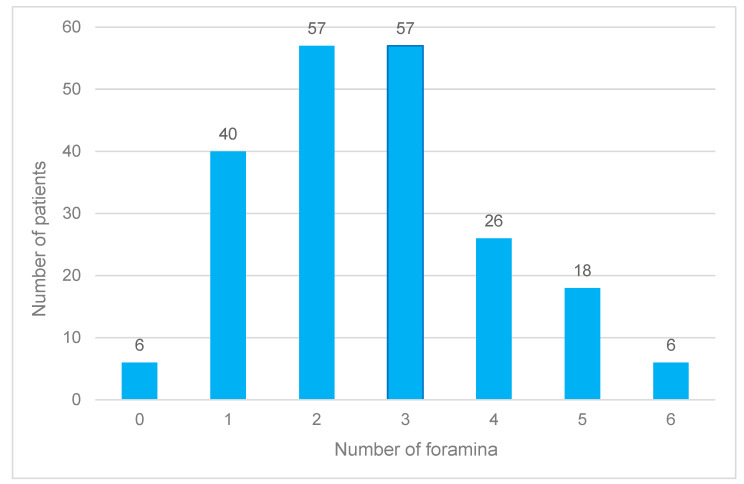
Frequency distribution of the accessory foramina of the canalis sinuosus across all age groups and independent of gender.

Men (51.5% of the subjects) had 307 foramina (55%), and women (48.5%) had 248 (45%). The most common number of foramina per patient was two or three (27.1% each). One, four, or five foramina occurred less frequently, while six or no foramina were observed in only 2.9% of participants (Figure 4).

On average, 2.6 foramina were recorded per patient, with men being affected more frequently (2.9 foramina) than women (2.3 foramina). This difference was significant. While at least one foramen was always detectable in men, no foramina were found in six women.

In terms of age, group 2 had the highest number of foramina, with an average of 2.8, followed by group 3 (2.5) and group 1 (2.6). These differences were not significant. The mean axial diameter across all ages was 0.9 mm, with women having smaller diameters on average (0.8 mm) than men (0.9 mm). The maximum axial diameter was 1.9 mm, with gender-specific variations: men showed larger maximum values than women, especially in group 1.

**Figure 5:**
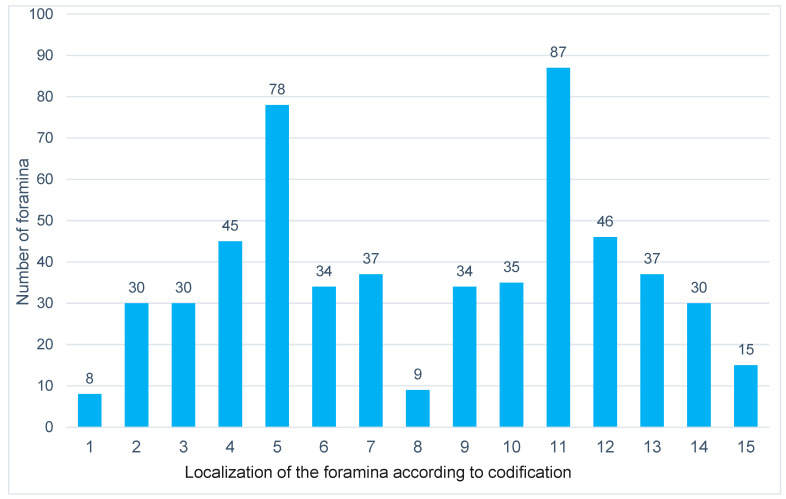
Representation of the distribution of the accessory foramina of the canalis sinuosus according to localization across all age groups and independent of gender. The foramen localization of numbers 1–15 is defined according to the described coding.

The most common location of the foramina was medio-palatal to tooth 22. Other common locations were medio-palatal to tooth 12 and proximal between teeth 22 and 23. Men more frequently had foramina close to tooth 22, while women more frequently had them medio-palatal to tooth 12 (Figure 5).

### 3.3. Analysis of Distance Measurements in the Axial and Sagittal Planes

The same patient population was used for distance measurements. The axial distance between the outer edge of a foramen and the pulp wall of the nearest tooth was 4.6 mm on average. On average, men had larger distances (4.8 mm) than women (4.5 mm). The distance increased with increasing age up to 60 years, but it stagnated in older men. The minimum axial distance was 2.1 mm in men and 2.0 mm in women. The maximum distance was 10.1 mm regardless of age, but it was lower in younger women at 7.1 mm.

In the sagittal plane, the average distance was 2.7 mm, with women showing slightly lower values. Older patients tended to have smaller maximum distances regardless of gender. Overlaps between the canal and tooth or the defined measuring line occurred in 75% of sagittal measurements, and they were more frequent in men than in women.

Of the 551 foramina, 30% were closer than 5 mm to the nearest tooth in the axial plane, and 21% were closer to the sagittal plane. The remaining four foramina could not be clearly localized. None of the measured variables showed significant age-specific or gender-specific differences.

### 3.4. Analysis of the Temporal Recordings in the Axial and Sagittal Planes

In the 10 patients examined in the third part of the evaluation, a total of 20 foramina were recognizable. Of these, 12 canal openings were observed in men (60%), while eight exit points were observed in female patients (40%). The mean diameter in male patients at the time of the first and second recording in the axial slice was unchanged at 1.0 mm. In contrast, this average value decreased in women from 0.9 mm to 0.8 mm. In the sagittal slice, the mean diameter increased by 0.1 mm over time in both sexes. In the case of the minimum diameter, no change was observed in women in either layer from the first to the second recording, while in men, this value increased by 0.1 mm in the sagittal plane.

The maximum diameter increased by 0.1 mm in the sagittal plane in women over time, while in men, this value decreased by 0.2 mm in the horizontal plane.

Female patients had one foramen less, with a diameter of more than 1 mm over time from the first to the second image, while no change was observed in the male participants.

A change in the diameter of more than 0.2 mm over time was more frequently observed in both planes in men (three vs. two) than in women (one vs. one).

However, no statistical significance could be determined for the changes over time listed here.

## 4. Discussion

Understanding the anatomical structures in the anterior maxilla is essential for dentists who perform surgery in order to avoid complications. For example, there is a risk of injury to neurovascular structures during implant surgery, which, in many cases, can result in increased bleeding or sensory impairment [19,20].

Digital volume tomography (CBCT) enables the precise visualization of bony structures in the surgical site through three-dimensional imaging and supports preoperative planning through the software-supported measurement of adjacent anatomical features [16,21]. This technology is of crucial importance not only for implant planning but also for endosurgical procedures or the removal of impacted teeth in the maxillary front in order to minimize intraoperative complications [21].

Since the canalis sinuosus and its accessory canals are often unknown in clinical practice, this study aimed to analyze their prevalence and changes over time in order to gain clinically relevant insights. In addition, the results of this study were compared with the current literature. The study by Machado et al. served as a reference and was supplemented by other studies, including those by Oliveira-Santos et al., von Arx et al., and Orhan et al. [2,4,5,7,8,9,22,23].

### 4.1. Discussion of Methods and Limitations

Digital volume tomography (CBCT) was used to examine the relevant bony structures, as it enables the precise visualization of the localization and course of accessory bone canals.

Two-dimensional images are unsuitable due to the small dimensions of the canals and the potential risk of confusion with inflammatory changes [24]. The device used (GXCB-500, Gendex Dental Systems, Hatfield, PA, USA) offers a resolution of 0.2 mm, which enables high-precision measurements. The seated positioning of the patients reduced movement artifacts and improved the image quality.

The present study has several methodological limitations that must be considered when interpreting the results. One significant limitation lies in the technical imaging method, particularly due to possible artifacts caused by long X-ray machine cycle times and metallic restorations in the beam’s path. These artifacts could impair the quality of the images and make it more difficult to identify and measure the accessory bone channels. Furthermore, the indication for CBCT was not determined within the scope of the study, meaning that the device settings varied depending on the original clinical question. As a result, some images could not optimally display the relevant anatomical structures, which limited the measurability of the canals.

Another methodological disadvantage is the lack of standardization with respect to image acquisition by different examiners. Despite standardized patient positioning, individual differences in image acquisition may have resulted in variations in image quality. Furthermore, the foramina were first detected in the axial plane and then confirmed in the sagittal plane, but the exact determination of the bony border for measuring the maximum diameter posed a challenge. This was especially true for the smallest foramina, which were difficult to detect due to the limited image resolution. The fact that no blinded control measurement was performed additionally increases the risk of inter-individually varying data collection and reduces the reproducibility of the results.

The measurement of the distances between the accessory foramina and the neighboring teeth or the buccal bone cortex was also subject to certain limitations. The selection of relevant measuring points could not be fully standardized because their recognizability varied in both planes. This opens up a certain freedom of interpretation such that measurement results could vary depending on the experience of the examiner.

In addition, particular methodological challenges arose when examining the temporal change in the number and diameter of the foramina in ten patients. Although the same X-ray machine was used for all measurement times, a variation in image quality due to different device settings, clinical questions, and indications is likely. Furthermore, reproducibility could have been affected by motion artifacts. Since no comparable study with such a long-term analysis has been published to date, there is also a lack of established reference values for validating the results.

In summary, the main limitations of the study arise from varying imaging conditions, potential artifacts, a lack of blinded control measurements, and the difficult standardization of measuring points. These factors could influence the precision of the results and should be considered when interpreting the data. 

### 4.2. Discussion of the Results

The results were analyzed by axial and sagittal plane as well as by age group and gender. Similar to the study by Shan et al. [8], foramina from a diameter of 1 mm were examined. A gender-specific comparison showed that men had significantly more foramina than women, although the differences in the mean values of the maximum diameters of the canals were small (men: 1.03 mm; women: 0.96 mm).

There were clear but not significant differences between the age groups. While the distances increased with increasing age in the axial plane, they decreased in the sagittal plane from the youngest to the oldest group. Changes in the number and diameter of the foramina over time could not be clearly demonstrated due to the small number of patients.

However, the study revealed clinically relevant findings for implant planning. Based on the average distances of 4.6 mm (axial) and 2.7 mm (sagittal), there was a high risk of the perforation of accessory canals when implants were placed in the anterior maxilla, especially when space was at a minimum. The implant’s diameter and position play a decisive role here. Safety distances of at least 1.5 mm to adjacent teeth and 3 mm to adjacent implants should be maintained.

### 4.3. Comparison with Current Studies

The prevalence of accessory canals is reported in the literature with a considerable range between 15.7% and 100% [25,26]. This variability is due to different examination methods, image resolutions, and inclusion and exclusion criteria. The present study found a prevalence of 97%, which is high compared to the results of other studies. This is due to the fact that smaller canals (<1 mm) were also included.

In their study, Machado et al. (2016) found at least one foramen of the canalis sinuosus in 52.1% of the examined patients [6]. In contrast, Wanzeler et al. reported a prevalence of 36.9%, whereby only canals with a diameter of at least 1 mm were examined [2]. Orhan et al. (2018) found a prevalence of 72.2% in women and 69.7% in men, which was also due to the inclusion of only larger canals [7]. These results make it clear that the recording method and the specified minimum size of the ducts have a considerable influence on prevalence data.

Another important feature of the present study is the gender-specific analysis. The results showed that men tended to have more foramina of the canalis sinuosus than women, which is also consistent with the studies of Orhan et al. and Machado et al. However, it should be noted that the differences between the sexes were significantly more pronounced, especially for larger foramina (>1 mm) [6,7].

With regard to the localization of the accessory canals, the results of the present study are largely consistent with the literature. Most studies report that the highest prevalence is in the vicinity of the central incisors or the lateral incisors. This was also confirmed in studies carried out by von Arx et al., Oliveira-Santos et al., and Wanzeler et al. [8,22,23]. The distances to neighboring teeth and the buccal cortical bone varied in different studies, whereby the results of the present study were within the existing variability.

A particularly noteworthy aspect is the comparison of the results with the study by Shan et al. (2021). They determined a prevalence of 36.9% in a comparable patient cohort, although a lower image resolution was used. This study confirms the assumption that higher image quality enables the more precise detection and measurement of smaller channels [8].

In addition to prevalence and localization, the change in the number and size of the accessory canals over time was also investigated. There are only a few studies on this to date, but they also point to an age-related increase in canal spacing. For example, both Beyzade et al. and Tomrukcu et al. reported an increase in spacing with increasing age, which can be attributed to progressive bone degeneration and remodeling [2,9]. Although the present study was able to confirm this observation, the number of patients per age group was too low to demonstrate significant differences.

Another relevant aspect is the clinical relevance of the results, especially with regard to implant planning. The identified safety margins of 4.6 mm on average in the axial plane and 2.7 mm in the sagittal plane should be taken into account when selecting the implant’s position and size. These results confirm the recommendations of Esposito et al. (1998), who suggested a safety distance of at least 1.5 mm to adjacent teeth and 3 mm to adjacent implants [27].

## 5. Clinical Relevance

The results underline the importance of precise preoperative planning, taking into account the neighboring anatomical structures. The use of navigated implantation techniques is particularly recommended where space is limited. Case reports show that complications due to perforations of the sinuosal canal can be avoided through careful planning and the selection of suitable implant lengths [28,29].

## Data Availability

The datasets used and/or analyzed during the current study are available from the corresponding author upon reasonable request.
